# Recombinant Production of Biliverdin IXβ and δ Isomers in the T7 Promoter Compatible *Escherichia coli* Nissle

**DOI:** 10.3389/fmicb.2021.787609

**Published:** 2021-12-08

**Authors:** Elizabeth A. Robinson, Nicole Frankenberg-Dinkel, Fengtian Xue, Angela Wilks

**Affiliations:** ^1^Department of Pharmaceutical Sciences, University of Maryland School of Pharmacy, Baltimore, MD, United States; ^2^Fachbereich Biologie, Abt. Mikrobiologie, Technische Universität Kaiserlautern, Kaiserslautern, Germany

**Keywords:** *E. coli* Nissle (EcN(T7)), *Pseudomonas aeruginosa*, HemO, heme, biliverdin IXβ, biliverdin IXδ, biliverdin IXα, biliverdin isomer

## Abstract

The ability to obtain purified biliverdin IX (BVIX) isomers other than the commercially available BVIXα is limited due to the low yields obtained by the chemical coupled oxidation of heme. Chemical oxidation requires toxic chemicals, has very poor BVIX yields (<0.05%), and is not conducive to scalable production. Alternative approaches utilizing recombinant *E. coli* BL21 expressing a cyanobacterial heme oxygenase have been employed for the production BVIXα, but yields are limited by the rate of endogenous heme biosynthesis. Furthermore, the emerging roles of BVIXβ and BVIXδ in biology and their lack of commercial availability has led to a need for an efficient and scalable method with the flexibility to produce all three physiologically relevant BVIX isomers. Herein, we have taken advantage of an optimized non-pathogenic *E. coli* Nissle (EcN(T7)) strain that encodes an endogenous heme transporter and an integrated T7 polymerase gene. Protein production of the *Pseudomonas aeruginosa* BVIXβ and BVIXδ selective heme oxygenase (HemO) or its BVIXα producing mutant (HemOα) in the EcN(T7) strain provides a scalable method to obtain all three isomers, that is not limited by the rate of endogenous heme biosynthesis, due to the natural ability of EcN(T7) to transport extracellular heme. Additionally, we have optimized our previous LC-MS/MS protocol for semi-preparative separation and validation of the BVIX isomers. Utilizing this new methodology for scalable production and separation we have increased the yields of the BVIXβ and -δ isomers >300-fold when compared to the chemical oxidation of heme.

## Introduction

**FIGURE 1:**
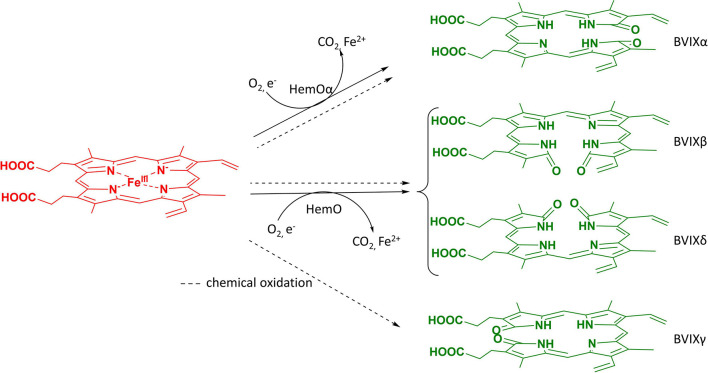
BVIX isomers produced by heme oxygenase or chemical oxidation. Heme oxygenases (HO’s) including mammalian and bacterial catalyze heme to BVIXα, CO_2_, and Fe^2+^. *P. aeruginosa* HemO degrades heme releasing the BVIXβ, and -δ isomers. All four isomers, including BVIXγ, are produced during the chemical oxidation reaction.

The catabolism of heme by the mammalian heme oxygenase (HO) produces biliverdin IXα (BVIXα) a metabolite well known for its anti-inflammatory and immunosuppressant functions through indirectly downregulating pro-inflammatory cytokines (TLR-4) (Bisht et al., 2014a) and upregulating anti-inflammatory cytokines (IL-10) (Bisht et al., 2014b) *via* biliverdin reductase (BVR) nitrosylation or phosphorylation, respectively. These modifications through subsequent signal transductions pathways lead to decreased TLR-4 expression and increased IL-10 production (Bisht et al., 2014a,b). In addition, BVIXα has anti-mutagenic (Arimoto et al., 1980; Bulmer et al., 2008) as well as anti-oxidant functions *via* the conversion of bilirubin IXα (BRIXα) to BVIXα and back *via* BVR (Stocker et al., 1987; Sedlak and Snyder, 2004; Baylor and Butler, 2019). HOs are seen across all kingdoms of life from bacteria to mammals where their main function is to break down heme into CO, BVIX metabolites, and iron for reutilization. Most HO’s have been found to produce the BVIXα isomer, except for the pathogen *Pseudomonas aeruginosa* whose heme oxygenase (HemO) degrades heme with the release of BVIXβ and -δ (Figure 1; Friedman et al., 2004). Recently there has been an increase in reports that other isoforms of BVIX, specifically BVIXβ and BVIXδ, play important physiological roles in eukaryotic and prokaryotic systems. In humans, BVIXβ is only seen during the first 20 weeks of gestation *via* the detection of bilirubin-IXβ in human fetal bile (Yamaguchi and Nakajima, 1995) and the expression of a human BVIXβ reductase (Yamaguchi et al., 1994; Pereira et al., 2001). While the degradation of heme to BVIXα by the mammalian HO’s (HO-1 and HO-2) has been well studied (Maines et al., 1986), the origin of the BVIXβ isomer is not as well understood. It is suggested that the production of BVIXβ may be the result of the switch from embryonic to fetal to adult hemoglobin during the birthing process (Pereira et al., 2001).

In the last two decades several bacterial pathogens have been shown to encode heme oxygenases and dioxygenases that degrade heme to release iron, a micronutrient required for survival and pathogenesis (Wilks and Heinzl, 2014; Wilks and Ikeda-Saito, 2014). In the opportunistic pathogen *P. aeruginosa* the iron regulated HemO oxidatively cleaves heme to release BVIXβ and BVIXδ (Ratliff et al., 2001). Heme uptake studies combined with LC-MS/MS analysis of the *P. aeruginosa hemO* deletion strain showed HemO is absolutely required to drive heme uptake into the cell (O’Neill et al., 2012). The significance of HemO in *P. aeruginosa* iron acquisition and its role in virulence has led to extensive study of the protein itself (Ratliff et al., 2001; Friedman et al., 2004). Additionally, recent studies by our lab have also shown the importance of HemO metabolites, BVIXδ and -β isomers, as signaling molecules in the post-transcriptional regulation of the *P. aeruginosa* heme sensing and acquisition pathways (Mourino et al., 2016). As heme acquisition has been shown to be the primary source of iron in *P. aeruginosa* chronic infection at the expense of iron siderophore systems (Marvig et al., 2014; Nguyen et al., 2014), HemO’s dual function in both sensing and uptake is clearly important for *P. aeruginosa* colonization and infection. Furthermore, targeting *P. aeruginosa* HemO has proven to be an effective therapeutic strategy by inhibiting both iron acquisition and the ability to sense extracellular heme (Liang et al., 2018; Centola et al., 2020; Robinson et al., 2021). Moreover, *P. aeruginosa* encodes a second BVIXα-dependent heme oxygenase, BphO, directly upstream of BphP, a sensor kinase as part of a two-component signaling system (Wegele et al., 2004; Barkovits et al., 2008). BVIXα functions as a far-red light chromophore for BphP and has been shown to be linked to the two component system KinB-AlgB which controls genes involved in biofilm formation (Mukherjee et al., 2019).

Currently, BVIXα is a commercially available compound produced by chemical oxidation of bilirubin sourced from mammalian bile (McDonagh and Palma, 1980), or by utilizing recombinant *E. coli* expression of HO from either cyanobacteria (Cornejo et al., 1998; Chen et al., 2012), rat (Ishikawa et al., 1991; Wilks and Ortiz de Montellano, 1993), or yeast (Michael and Pendrak, 2005). A scalable bioreactor method utilizing a cyanobacterial HO showed enhanced production and comparable purity to commercial BVIXα, however, the method is limited by the rate of endogenous heme biosynthesis (Chen et al., 2012). Furthermore, recombinant expression systems to date have been limited to the production of BVIXα. For the past 80 years, chemical oxidation of heme (McDonagh and Palma, 1980) and the oxidation of bilirubin IXα to BVIXα(McDonagh and Assisi, 1971; Elich et al., 1989) have been the main methods for producing all four BVIX isomers (Figure 1). While there has been some improvement in the production and separation of the four isomers, through preparative TLC methods of the esterified BVIX isomers (Heirwegh et al., 1991) to high-performance liquid chromatography (HPLC) methods of the BVIX isomers (Barker et al., 2012; Robinson et al., 2021), the overall yields especially those of BVIXβ and -δ are very low (Zhang et al., 2020). Recently, Zhang et al., (2020) described the scalable recombinant expression of the *P. aeruginosa* HemO as a means of obtaining BVIXβ and BVIXδ, however, the system is again limited by the rate of endogenous heme biosynthesis. Herein we report on a scalable BVIX production method that exploits the over expression of *P. aeruginosa* HemO in a recently engineered strain of the non-pathogenic probiotic (O6:K5:H1) *E. coli* strain Nissle 1917 (EcN) (Lodinova-Zadnikova et al., 1998; Rembacken et al., 1999; Sonnenborn, 2016). The EcN genome encodes an endogenous heme receptor, ChuA, which was shown to increase exogenous heme uptake into the cell (Fiege et al., 2018). This strain was further optimized by integrating a T7 RNA polymerase gene into the chromosome of EcN (EcN(T7)) for use with a wide array of T7-promoter expression vectors (Fiege and Frankenberg-Dinkel, 2020). The combination of an outer membrane receptor ChuA for heme uptake and a T7 RNA polymerase for over-expression of genes make this an ideal strain for the production of fully reconstituted heme proteins. Additionally, the strain can be used to express or co-express genes to produce heme metabolites such as the BVIXs or related linear tetrapyrroles. Herein, we have exploited the EcN(T7) strain for the over-expression of the *P. aeruginosa* HemO as a means of generating BVIXβ and BVIXδ on supplementation of the cultures with exogenous heme. Additionally, over expression of the BVIXα producing HemO N19K/K34A/ F117Y/K132A mutant (Barker et al., 2012; Heinzl et al., 2016) (here on referred to as HemOα) allowed for production of BVIXα by a similar extraction and purification method for direct comparison purposes.

Additionally, we have improved upon the biliverdin extraction and HPLC protocol for the separation of the BVIX isomers utilizing our recently optimized LC-MS/MS assay (Dent and Wilks, 2020). This method to produce the BVIXβ and -δ isomers give a >300-fold increase in yield of the individual isomers when compared to the chemical coupled oxidation of heme while avoiding the use of toxic chemicals. Furthermore, the EcN(T7) strain has several advantages over other reported expression systems, namely the ability to feed heme to the cells side stepping the rate limiting step of heme biosynthesis and the scalable nature of the system for high-volume bioreactor systems.

## Methods

### Reagents and Materials

All glassware and metalware were cleaned in a solution of 0.1 M NaOH and 1% SDS, rinsed with MilliQ water, autoclaved at 550°C. Precautions to avoid contaminants within the BVIX isomer extraction and HPLC separation were taken using HPLC grade reagents and solvents (Fisher Optima). Similar precautions for LC-MS/MS BVIX isomer validation using LC-MS grade (Fisher Optima and Thermo Scientific for DMSO) reagents and solvents. Specific medias, columns, and instruments are listed below where appropriate.

### Biliverdin IX Isomer Production *via* Heme Oxygenase Over-Expression in *Escherichia coli* Nissle (T7)

*Escherichia coli* Nissle(T7) cells were obtained from the Frankenberg-Dinkel laboratory. EcN(T7) electrocompetent cell were made fresh as previously described (Dower et al., 1988) with some modifications. Briefly, 5 mL LB (Lennox) cultures inoculated with EcN(T7) were shaken at 210 rpm overnight at 37°C in 10 mL culture tubes. The overnight culture was diluted into fresh 25 mL LB cultures in a 125 ml flask to a final OD_600_ = 0.05 and grown with shaking (210 rpm) at 37°C until the OD_600_ reached 0.5–0.6 (mid-log phase). The culture was then centrifuged at 5,000 × *g* at 4°C for 5 min and pellets were washed with 25 mL chilled 10% glycerol and the wash repeated twice more. Following the washing steps the culture was recentrifuged and suspended in 250 μL of 10% glycerol to a final 100x lower volume from the initial culture. 30 μL aliquots of the final cell volume (250 μL) were used for electroporation or stored at −80°C for up to 3 months. Electroporation was performed as previously described (Miller and Nickoloff, 1995) with slight modifications. Briefly, 1–5 μL of pET*hemO* or pET*hemO*α (Ratliff et al., 2001; Heinzl et al., 2016; Liang et al., 2018; Robinson et al., 2021), was electroporated into electrocompetent EcN(T7) cells using prechilled 2 mm gap electroporation cuvettes and a BioRad Micropulser Electroporator on the “Bacteria: Ec1” setting (1.8 kV, 1 pulse). 1 mL SOC media at 25°C was immediately added to the electroporated culture, transferred to an ice-cold culture tube, and set to shake at 210 rpm for at least 1 h at 37°C. 250 μL of electroporated cells were then plated onto LB agar containing a final concentration of 100 μg/mL ampicillin and incubated overnight at 37°C. The resulting colonies were inoculated into 25 mL LB media containing 100 μg/mL ampicillin and shaken at 250 rpm overnight at 37°C. HemO overexpression was performed as previously described (Ratliff et al., 2001; Heinzl et al., 2016; Robinson et al., 2021) with slight modification to increase aeration and optimize HemO expression. Briefly, an overnight culture was diluted into fresh 100 mL of LB media containing 100 μg/mL ampicillin in 250 mL baffled flasks and shaken at 250 rpm for 1.5–2 h at 37°C or until they reached an OD_600_ = 0.4–0.5. Cultures were induced with 1 mM IPTG and shaken at 250 rpm for a further 2 h at 25°C. The cultures were then supplemented with appropriate concentrations of heme (10, 15, 20, and 25 μM) prepared in DMSO (10–100 μL) and shaken at 250 rpm overnight at 25°C. Cultures were pelleted (5,000 × g, 20 min, 4°C) in 50 mL conical tubes, and the supernatants were collected for BVIX extraction or stored at −80°C for up to 1 month. Pellets were thawed, resuspended in Milli Q water and 2 μg/mL DNase I, sonicated at 100% amplification for 5 secs on and 15 secs off for 2 min, and run on a 12% SDS PAGE gel at 200V for 30 min as previously described with slight modifications (Heinzl et al., 2016; Liang et al., 2018; Robinson et al., 2021).

### Extraction of Biliverdin IX Isomers From *Escherichia coli* Nissle(T7) Supernatants

Biliverdin IX isomers were extracted as previously described (Mourino et al., 2016) with slight modification. Briefly, supernatants were filtered through a 0.2 μm PVDF membrane to remove any remaining particulate matter. In the dark, supernatants were acidified to pH 2.5 with 10% TFA, and loaded over a C18 Sep-Pak column (35cc, Waters) equilibrated with 20 mL each of acetonitrile (ACN), H_2_O, 0.1% TFA in H_2_O, and methanol:0.1% TFA (10:90). After sample application, the column was washed with 40 mL 0.1% TFA, 40 mL ACN:0.1% TFA (20:80), 20 mL methanol:0.1% TFA (50:50) and eluted with 15 mL methanol. Alternatively, for smaller batches the isomers were purified over a 3cc (Waters) C18 Sep-Pak column equilibrated as above scaling back the washes and elution volumes x10. The supernatant was centrifuged (14,800 rpm, 5 min, 25°C) and the remaining solution containing purified BVIX isomers was collected. Purified BVIX isomers were diluted with an equal volume of water and extracted into CHCl_3_ (3–5 ml) washed with ×3 with 5 ml H_2_O to remove any remaining acid. The CHCl_3_ layer was collected, and speed vacuumed dry and stored at −80 °C prior to HPLC separation.

### HPLC Separation of Biliverdin IX Isomers

Dried BVIX aliquots were resuspended in 100 μL HPLC-grade methanol and centrifuged (14,800 rpm, 5 min, 25°C) to remove any particulates. The BVIX isomers were then separated by HPLC (Thermo Fisher Ultimate^TM^ 3000 HPLC) on an Synergi^TM^ 4 μm Fusion-RP 80 Å C18 Column (250 × 10 mm) at a flow rate of 3 mL/min. The mobile phase consisted of solvent A, H_2_O:0.1% formic acid, and solvent B, ACN:0.1% formic acid. A linear gradient was run starting at 64% A and 36% B and ending with 0% A and 100% B over 20 or 30 mins. BVIX isomer retention times were 9.9, 10.8, and 11.6 min for BVIXα, BVIXδ, and BVIXβ isomers, respectively, for the 20 min linear gradient and 9.7, 12.1, and 13 min, respectively for the 30 min linear gradient. Separated isomers were then extracted into choloroform as described above, speed vacuumed dry and stored at −80°C. The final yield of each BVIX isomer was calculated from their respective millimolar extinction coefficients (ε_*mM*_) at 376 nm and 665 nm in methanol as previously described (Heirwegh et al., 1991). BVIXα, -β, and -δ millimolar extinction coefficients at 376 nm are 50.6, 51.7, and 47.1 mM^–1^ cm^–1^, respectively, and at 665 nm are 14.6, 15.4, and 15.5 mM^–1^ cm^–1^, respectively. Yields were calculated using Eq. 1 below:


(1) $$\frac{T\,o\,t\,a\,l_{E}}{T\,o\,t\,a\,l_{C}}\times 100=P\,e\,r\,c\,e\,n\,t\,Y\,i\,e\,l\,d$$

Total_C_ represents the total μmols of BVIX calculated to give 100% yield and Total_*E*_ represents the total μmols of BVIX obtained experimentally. For the BVIX production method by coupled oxidation Total_C_ for the individual BVIX isomers was calculated based on 25% of the total BVIX yield, while the HemO EcN(T7) BVIX production method, Total_C_ for BVIXβ, and -δ isomers were calculated based on HemO BVIX ratio of 30:70, respectively. Molar extinction coefficients of BVIXα, -β, and -δ isomers in DMSO were obtained using 380 ± 1/658 ± 3 nm, 389 ± 1/651 ± 1 nm, and 386 ± 2/645 ± 5 nm, respectively, (*n* = 3) based on the UV spectrum (Supplementary Figure 5). Extinction coefficients (ε) of BVIXα, -β, and -δ isomers in DMSO were calculated to be 20 ± 0.2/ 4.9 ± 0.2 mM^–1^ cm^–1^, 35.9 ± 12.0/ 9.0 ± 4.0 mM^–1^ cm^–1^, and 11.5 ± 5.3, 2.9 ± 1.4 mM^–1^ cm^–1^, respectively (Supplementary Figure 5).

### LC-MS/MS Analysis of Biliverdin IX Isomers

Following HPLC separation the respective BVIX isomers were confirmed by LC-MS/MS as previously described with slight modification (Dent and Wilks, 2020). Briefly, the purified BVIX isomers were resuspended in 10 μL DMSO, diluted to 100 μL with mobile-phase ACN:H_2_O (50:50, v/v), and centrifuged at 14,000 rpm for 5 min at 4°C to remove particulates. BVIX isomers (2 μL) were analyzed on a Waters TQ-XS triple quadrupole mass spectrometer with AQUITY H-Class UPLC fitted with an Ascentis RP-amide 2.7-mm C18 column (10 cm × 2.1 mm) at 30^*o*^C with a flow rate of 0.4 mL/min. The mobile phase consisted of solvent A, H_2_O:0.1% formic acid, and solvent B, ACN:0.1% formic acid. The initial gradient was 64% A and 36% B followed by 5 min at 55% A and 45% B, 8 min at 40% A and 60% B, 8.5 min at 5% A and 95% B, and 10 min 64% A and 36% B. Using multiple-reaction monitoring (MRM), the BVIX precursor ions were detected at 583.21 and the major ions following fragmentation for BVIXα, BVIXβ, and BVIXδ isomers were 209.2/296.6, 343.1, and 402.2 m/z, with collision energies of 38, 36, and 30 V, respectively. The source temperature was set to 150°C, the capillary voltage to 3.60 kV, and the cone voltage to 43 V. Purity of the BVIX isomers was determined by the ratio of the integration of the isomer peaks to the total integration of the LC chromatograms.

## Results

### Optimization of Biliverdin IX Production *via* Heme Oxygenase Expression in *Escherichia coli* Nissle(T7)

**FIGURE 2:**
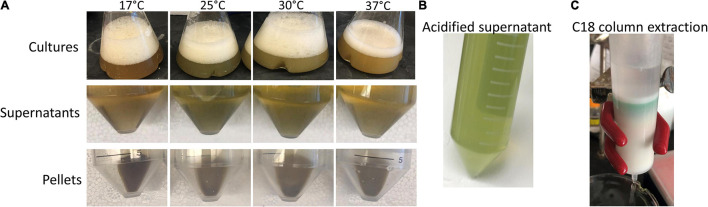
Induction temperature optimization of HemO over-expression in EcN(T7) cells. **(A)** Cultures, supernatants, and pellets after 16 h of induction at different temperatures (17, 25, 30, and 37) shaking at 250 rpm, supplemented with 10 μM heme, and induced with 1 mM IPTG. **(B)** Acidified supernatant. **(C)** Acidified and filtered supernatant applied onto a C18 Sep-Pak column (35cc, Waters) showing a green band of BVIXδ and BVIXβ isomers.

Expression of HemO in EcN(T7) was performed across a range of induction temperatures, incubation times, and shaking conditions (Figure 2). The optimal temperature and incubation time for maximum production of BVIX was found to be 25 °C for 16 h with 250 rpm shaking as judged by the green pigmentation in the supernatant (Figure 2). We found increased shaking improved BVIX yields as the increased oxygenation aided HemO-dependent heme degradation. Similarly, we tested a range of heme concentrations (10, 15, 20, and 25 μM) and found 10 μM heme gave the highest BVIX yield whereas higher concentrations led to a decrease in growth rate, most likely a result of heme toxicity. The expression of HemOα in EcN(T7) was slightly lower than that of HemO as judged by SDS PAGE (Supplementary Figure 4). The reason for the decrease in expression of HemOα is not clear as this was not previously observed on expression of HemO in *E. coli* BL21(DE3) (Mourino et al., 2016).

### Biliverdin IX Isomers Extraction and Purification

**FIGURE 3:**
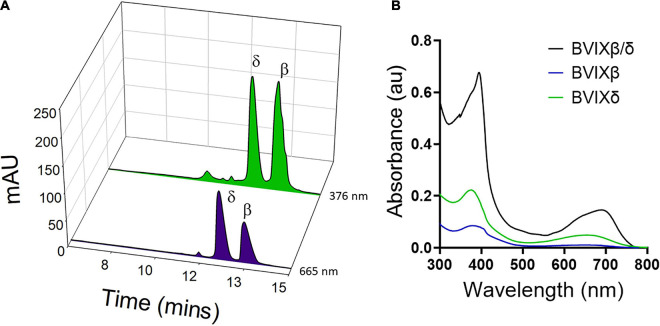
HPLC separation of BVIXδ, and -β isomers from supernatant. **(A)** HPLC separation of extracted BVIXδ and -β isomers using a linear gradient of solvent A, ACN:0.1% formic acid and B, H_2_O:0.1% formic acid over 30 min with retention times 12.1 min and 13.0 min, respectively. **(B)** UV-vis spectrum of C18 purified BVIXβ/δ (peaks at 394 and 680 nm) and HPLC purified BVIXβ (peaks at 378 and 650 nm), and -δ isomers (peaks at 376 and 650 nm) in 100% MeOH.

**FIGURE 4:**
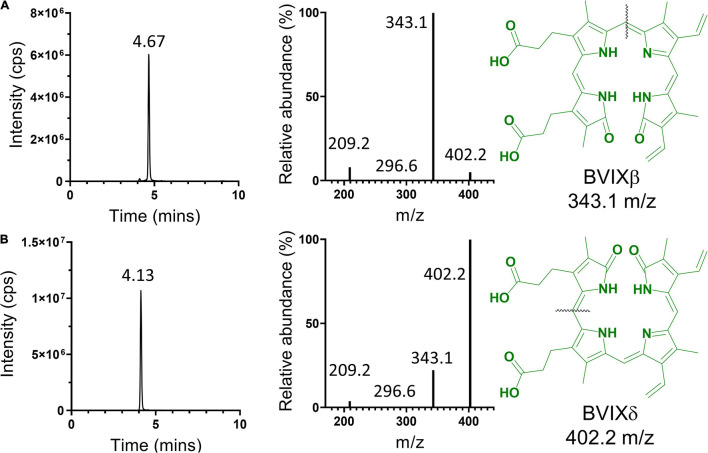
LC-MS/MS analysis of HPLC separated BVIX isomers. BVIXβ **(A)**, and -δ **(B)** isomers with LC retention times of 4.67 and 4.13 min, respectively. BVIXβ **(A)**, and -δ **(B)** isomers with majority precursor ions of 343.1, and 402.2 m/z, respectively. Black lines represent the fragmentation pattern for the respective isomers.

The C18 BVIX isomer extraction was performed in semi-darkness to protect the isomers from photoisomerization. The C18 eluted BVIX fraction was centrifuged to remove any excess acidified proteins and the UV-visible spectrum of the fraction was recorded. The absorption spectrum while characteristic of BVIX with a broad absorbance at 680 nm (Heirwegh et al., 1991) did appear to contain some contaminants most likely heme, as judged by the peak at 400 nm and the shoulder at 630 nm. (Figure 3B). Following HPLC purification (Figure 3A) the separated BVIXβ and -δ isomers were analyzed by absorption spectroscopy (Figure 3B) and LC-MS/MS (Figure 4). The absorption spectrum of the purified BVIXβ, and -δ isomers showed peaks at 378 and 650 nm, and 376 and 650 nm, respectively (Figure 3B). The BVIXα from the over-expression of HemOα in EcN(T7) was similarly purified and analyzed as shown in the Supplementary Information (Supplementary Figures 2, 3). Purified BVIXα yielded an absorption spectrum with peaks at 374 and 650 nm (Supplementary Figure 2B). The resulting purified BVIX isomers were extracted into chloroform washed, speed vacuum dried and stored in the dark at −80°C.

Our newly optimized HPLC method using a linear gradient consisting of solvent A, H_2_O:0.1% formic acid, and solvent B, ACN:0.1% formic acid can achieve baseline separation of the BVIXβ and -δ isomers, (Figure 3A). Comparing the yields between the BVIXβ and -δ isomer production by over expression of HemO in EcN(T7) (26 and 24%, respectively) to the chemical coupled oxidation (0.035 and 0.054%, respectively) shows a >700-fold and >400-fold increase, respectively (Table 1). The yields of BVIXα from the over expression of HemOα in EcN(T7) (1.4%) are lower than that of HemO WT but still >15-fold higher than the yield from the chemical coupled oxidation (0.09%) (Table 1).

**TABLE 1:** BVIXβ, and -δ yields from coupled oxidation and HemO EcN(T7) BVIX production methods.

Chemical coupled oxidation	Total_*C*_ (μ mols)	Total_*E*_ (μ mols)	Yields (%)
Heme	405	n.a.	n.a.
BVIXβ	108	0.038 ± 0.006	0.035 ± 0.006
BVIXδ	108	0.059 ± 0.002	0.054 ± 0.002
**HemO EcN(T7)**			
Heme	1	n.a.	n.a.
BVIXβ	0.3	0.079 ± 0.032	26 ± 10
BVIXδ	0.7	0.17 ± 0.030	24 ± 4.3

*The results are the average of three independent experiments. See the section “Methods” for calculations.*

### LC-MS/MS Validation of Biliverdin IX Isomers

The HPLC purified BVIXδ and -β isomers were further validated by LC-MS/MS and shown to have >99% purity based on the fragmentation patterns (Figure 4). Fragmentation and MRM of the BVIX precursor ion (583.21 m/z) yielded ions for BVIX-δ and -β isomers at 402.2 and 343.1 m/z, respectively (Figure 4). Similarly, for BVIXα the purity was >99% as judged by the major ion at 209.2 m/z (Supplementary Figure 3).

## Discussion

Previous work utilizing HemO over-expression in *E. coli* BL21 (DE3) has been used as a means of producing BVIXδ and BVIXβ (Zhang et al., 2020). Similarly, in our own work the *E. coli* BL21 (DE3) strain was employed to produce BVIXδ and BVIXβ, but the yields obtained were lower than that of the chemical coupled oxidation of heme (<0.05%), even when supplementing cultures with exogenous heme or the heme precursor δ-aminolevulinic acid to promote heme biosynthesis. Given the ability of the engineered EcN(T7) to naturally transport heme we coupled this to the over expression of HemO in an attempt to maximize BVIX yields. Interestingly, following centrifugation of the cultures, we saw no green coloration in the pellet as was observed on HemO expression in *E. coli* BL21 (DE3) (Wilks, 2003; Zhang et al., 2020). However, the green pigmentation in the supernatant, especially after acidification (Figures 2A,B), suggests that EcN(T7) cells may excrete the BVIX isomers more efficiently (Figure 2A and Supplementary Figure 1). We have previously shown *P. aeruginosa* (PAO1) also secretes BVIXα into the supernatant (Mourino et al., 2016), suggesting that bacteria such as EcN(T7) and PAO1 that acquire extracellular heme may also have a mechanism to secrete the BVIX metabolites. The secretion of the BVIX isomers proved advantageous by eliminating the necessity to lyse the cell pellet and denature HemO to release the BVIX isomers, as was necessary when expressing the protein in *E. coli* BL21 (DE3) strain where the majority of BVIX remains in the cell pellet (Supplementary Figure 1). The same lack of green coloration was observed in pellets following over-expression of HemOα. However, while BVIXα yields increased compared to the chemical oxidation of heme, they were significantly lower than the BVIXβ and -δ isomers produced by HemO WT, presumably due to the decreased HemOα protein levels compared to HemO (Supplementary Figure 1). Interestingly, we have not observed differences in the protein levels of HemO and HemOα in *E. coli* BL21 (DE3) cells (Mourino et al., 2016). Furthermore, following purification of HemO and HemOα, CD analysis showed both proteins have a similar overall structural fold and thermal stability profile (Mourino et al., 2016). Therefore, further optimization of the EcN(T7) expression system will be required for scale up of BVIXα production by either optimizing HemOα protein levels or employing an alternative bacterial or mammalian BVIXα-selective heme oxygenase. However, while the current yields on a per liter basis were lower (5–10 fold) than that previously reported for production of BVIXα in *E. coli* BL21(DE3), it should be noted that the authors utilized a modified cyanobacterial *ho1* gene and performed the expression in a large scale Bioreactor (Chen et al., 2012). Furthermore, in the current protocol we employed an extra HPLC purification step following extraction and semi-purification by C18 chromatography. However, while the production of BVIXα in EcN(T7) systems can be further optimized the main goal of the current work was to develop a method to produce the non-commercially available BVIXβ and -δ isomers by a method other than chemical coupled oxidation. Compared to the chemical coupled oxidation of heme, this approach provides a greener alternative to obtain BVIXβ and -δ that no longer uses harsh chemicals such as pyridine and decreases the total chloroform (CHCl_3_) extraction steps of which both are toxic and carcinogenic (National Toxicology Program, 2000; Meek et al., 2002).

In addition to optimizing production of the BVIXδ and -β isomers, we further improved on the HPLC purification by optimizing a method that allows for baseline separation of the BVIXβ, and -δ isomers utilizing a linear gradient of ACN 0.1% formic acid and H_2_O 0.1% formic acid. Previous ACN-based HPLC methods for the separation of the BVIX isomers have reported baseline separation of BVIXα from the BVIXδ and -β isomers, but no baseline separation between the BVIXδ and -β isomers (Yamaguchi et al., 1979). Baseline separation of the BVIXδ and -β isomers by HPLC has been previously reported with a mobile phase consisting of 20 mM formic acid in acetone (Barker et al., 2012; Zhang et al., 2020). However, the use of such a mobile phase is not ideal given the increased polarity and volatility when compared to ACN leading to inconsistent retention times between HPLC runs.

## Conclusion

Chemical oxidation heme or the oxidation of bilirubin have been the primary methods to obtain all four BVIX isomers or BVIXα, respectively. While the predominant role of BVIXα in mammalian physiology led to its commercial production, the growing awareness of the role of the BVIXδ and -β isomers particularly in bacterial systems has led to the need for alternative and greener methods to produce all three BVIX isomers. The method developed herein utilizing HemO’s unique regioselectivity coupled with the optimized EcN(T7) *E. coli* strain provides a cost effective and less toxic approach to obtaining semi-preparative quantities of the physiologically relevant BVIX isomers. This method increases the BVIXδ and -β isomer yields from <0.05 to >20%, a 400-fold increase. We have improved the HPLC separation process to include more widely acceptable and less harsh solvents. Furthermore, we have developed a bioreactor compatible bacterial expression system by which to produce all three physiologically relevant BVIX isomers and more importantly eliminate the use of harsh chemicals.

## Data Availability Statement

The authors acknowledge that the data presented in this study must be deposited and made publicly available in an acceptable repository, prior to publication. Frontiers cannot accept a manuscript that does not adhere to our open data policies. The data presented in the study are deposited in the MetaboLights repository, accession number MTBLS3555.

## Author Contributions

ER and AW were involved in the initial concept and design of the experiment as well as the acquisition and analysis of the data. ER, AW, NF-D, and FX were involved in the subsequent experimental design and data interpretation, and the drafting and revising of the manuscript for publication. All authors contributed to the article and approved the submitted version.

## Conflict of Interest

The authors declare that the research was conducted in the absence of any commercial or financial relationships that could be construed as a potential conflict of interest.

## Publisher’s Note

All claims expressed in this article are solely those of the authors and do not necessarily represent those of their affiliated organizations, or those of the publisher, the editors and the reviewers. Any product that may be evaluated in this article, or claim that may be made by its manufacturer, is not guaranteed or endorsed by the publisher.
