# Supplementary material for: Recombinant Production of Biliverdin IXβ and δ Isomers in the T7 Promoter Compatible Escherichia coli Nissle
Source: Front Microbiol. 2021 Dec 8;12:787609. doi: 10.3389/fmicb.2021.787609 (PMC8692735; doi:10.3389/fmicb.2021.787609)
Supplement: Supplementary file 1 [file Data_Sheet_1.PDF]

Supplementary Information

Recombinant production of biliverdin IXβ and δ isomers in the T7 promoter compatible *Escherichia coli* Nissle (EcN(T7))

Elizabeth A. Robinson[1], Nicole Frankenberg-Dinkel[2], Fengtian Xue[1], Angela Wilks[1]*

[1]Department of Pharmaceutical Sciences, University of Maryland Baltimore, Baltimore MD 21201, United States

[2]Fachbereich Biologie, Abt. Mikrobiologie, Technische Universität Kaiserlautern, Erwin-Schrödinger-Straße 56, D-67663 Kaiserslautern, Germany

*Corresponding Author Email: awilks@rx.umaryland.edu

Table of Contents:

Figure S1. HemOα EcN(T7) production of BVIXα culture, pellet, and C18 column extraction……...Page S2

Figure S2. BVIXα HPLC separation, absorption spectra, and LC-MS/MS analysis…………………..Page S3

Figure S3. LC-MS/MS analysis of BVIXα isomer……………………………………………………..Page S4

Figure S4. SDS PAGE gel of HemO and HemOα expression in EcN(T7) and BL21 (DE3)……….....Page S5

Table S1. Comparison of BVIXα isomer production…………………………………………………..Page S6

Figure S5. BVIX isomers molar extinction coefficients in DMSO……………………………….…Page S7

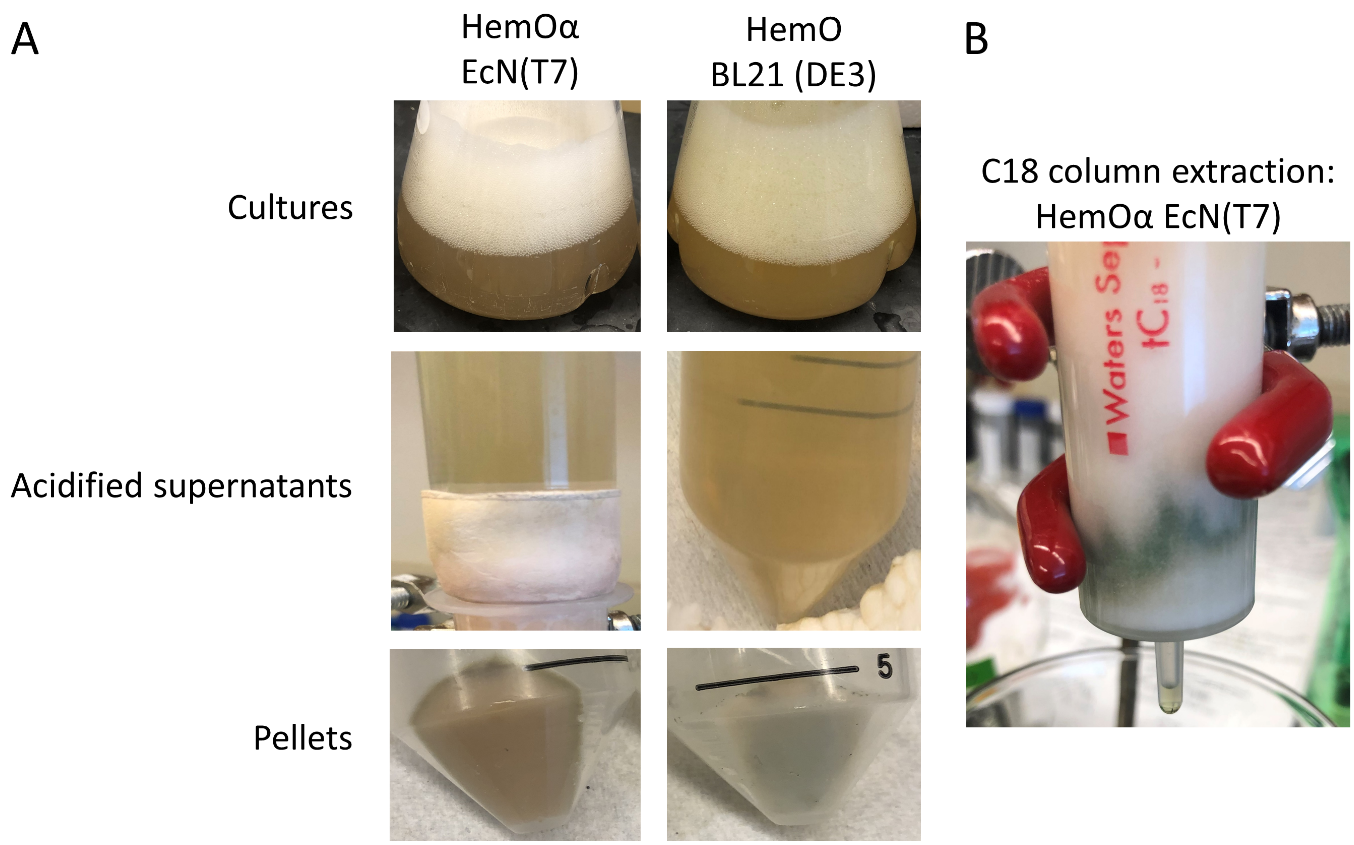

Figure S1. HemOα over expression in EcN(T7) cells and HemO over-expression in BL21 (DE3). (A) Cultures, acidified supernatants, and pellets after 16 h of induction at 25 °C shaking at 250 rpm, supplemented with 10 µM heme, and induced with 1mM IPTG. Comparison of filtered and acidified supernatants of HemO and HemOα over expression show green coloration. **(B)** Filtered and acidified supernatant applied onto a C18 Sep-Pak column (35cc, Waters) showing a green band of BVIXa isomer.

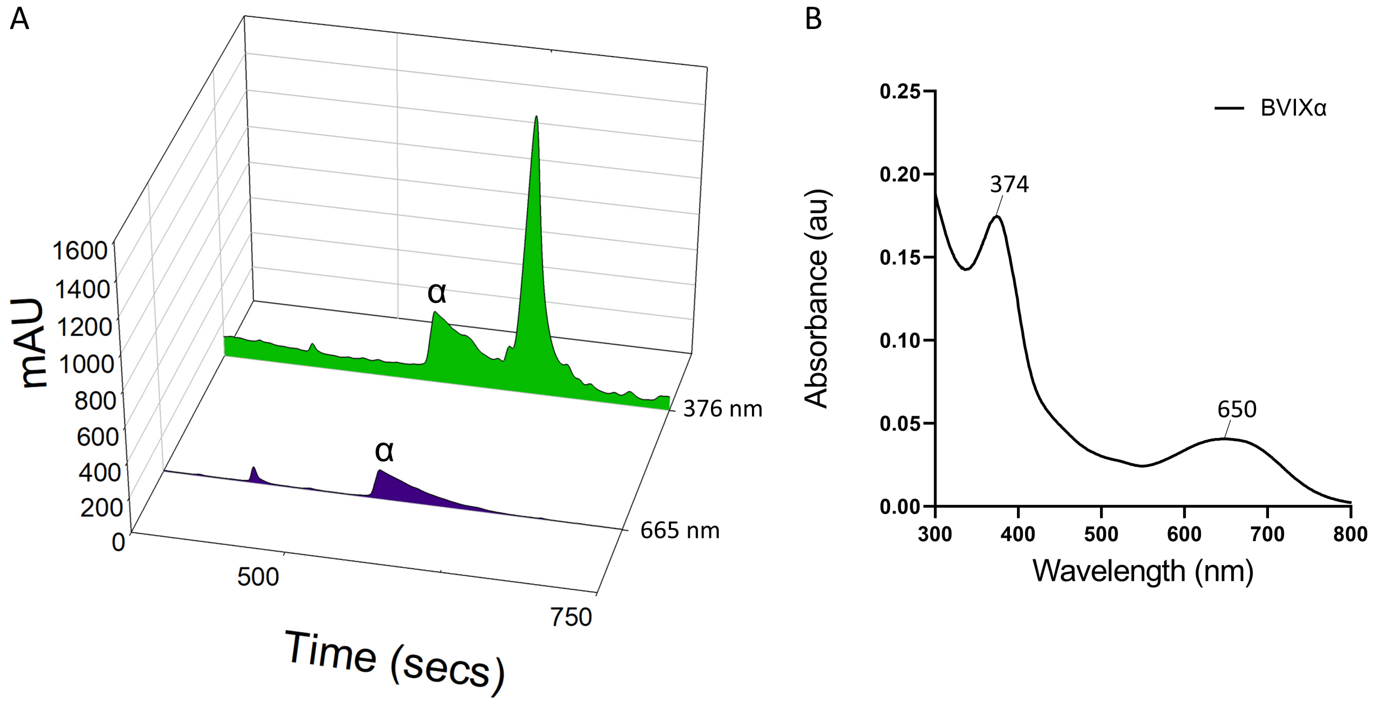

Figure S2. BVIXα HPLC separation and absorption spectra. (A) HPLC separation of extracted BVIXα using a linear gradient of solvent A, ACN:0.1% formic acid and B, H_2O:0.1% formic acid over 30 mins with retention time of 9.7 mins. **(B)** UV-vis spectrum of HPLC purified BVIXα in 100% MeOH with peaks at 374 nm and 650 nm.

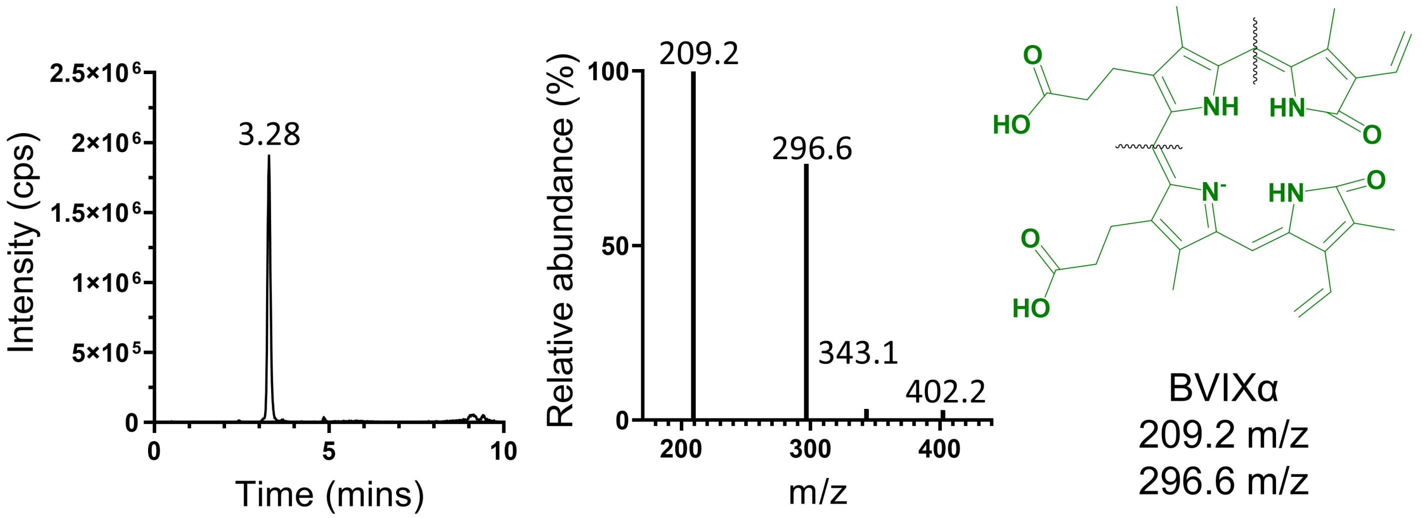

Figure S3. LC-MS/MS analysis of BVIXα isomer. LC-MS/MS analysis of HPLC separated BVIXα. BVIXα with LC retention times of 3.28 mins. BVIXα with majority precursor ions of 209.2 and 296.6 m/z. Black lines represent fragmentation pattern.

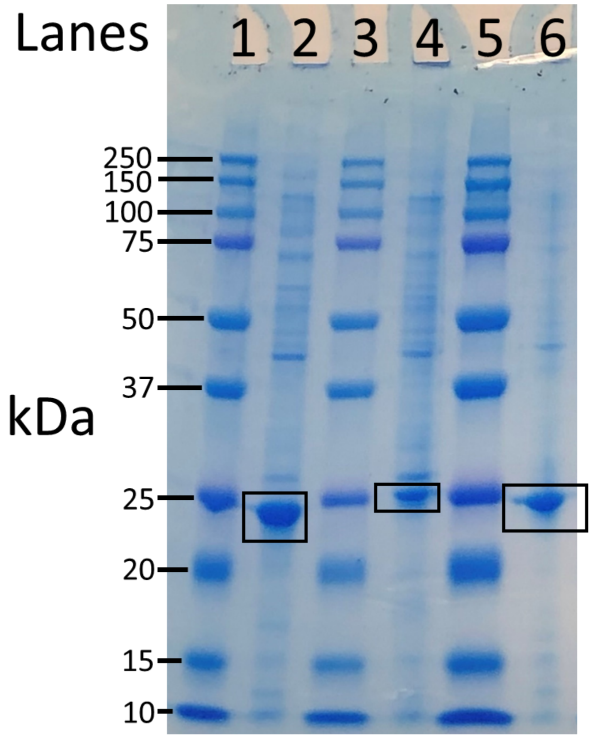

Lanes	Content
1	Protein ladder
2	HemO in EcN(T7)
3	Protein ladder
4	HemOα in EcN(T7)
5	Protein ladder
6	HemO in BL21 (DE3)

Figure S4. SDS PAGE gel of HemO and HemOα expression in EcN(T7) and BL21 (DE3). Cultures expressing HemO and HemOα in EcN(T7) and BL21 (DE3) cells were pelleted for BVIX extraction of the supernatants. Samples were prepared in water and run on a 12% SDS PAGE gel at 200V for 30 mins. HemO and HemOα protein bands are seen at ~22 kDa in both EcN(T7) and BL21 (DE3) pellets.

**Chemical Coupled Oxidation**	**Total$_C$ (µmols)**	**Total$_E$ (µmols)**	**Yields (%)**
Heme	405	n.a.	n.a.
BVIXα	108	0.1	0.09
**HemO EcN(T7)**			
Heme	1	n.a.	n.a.
BVIXα	1	0.014	1.4

Table S1. Comparison of BVIXα isomer production. BVIXα yields from chemical coupled oxidation (0.09%) and HemOα over-expression in EcN(T7) BVIX production (1.4%) methods. See Methods and Materials Eq. 1 for calculation.

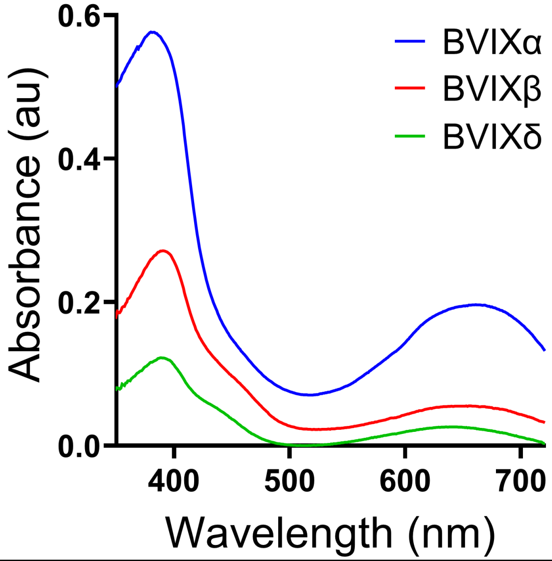

BVIX isomers

	λ_1 (nm)	ε_1 ($mM^{-1}\ cm^{-1}$)	λ_2 (nm)	ε_2 ($mM^{-1}\ cm^{-1}$)
α	380 ± 1	20.0 ± 0.2	658 ± 3	4.9 ± 0.2
β	389 ± 1	35.9 ± 12.0	651 ± 1	9.0 ± 4.0
δ	386 ± 2	11.5 ± 5.3	645 ± 5	2.9 ±1.4

Figure S5. BVIX isomers molar extinction coefficients in DMSO. Purified BVIXα, -β, and -δ isomers UV spectrum in DMSO showed peaks at 380 ± 1/ 658 ± 3 nm, 389 ± 1/ 651 ± 1 nm, and 386 ± 2/ 645 ± 5 nm, respectively (n = 3). BVIXα, -β, and -δ isomers molar extinction coefficients were found to be 20 ± 0.2/ 4.9 ± 0.2 $mM^{-1}\ cm^{-1}$, 35.9 ± 12.0/ 9.0 ± 4.0 $mM^{-1}\ cm^{-1}$, and 11.5 ± 5.3, 2.9 ± 1.4 $mM^{-1}\ cm^{-1}$, respectively.
